# Effects of a Digital Decision Support System on Diabetes Management in a Real-World Home Care Setting: Prospective Quasi-Experimental Feasibility and Implementation Study

**DOI:** 10.2196/89687

**Published:** 2026-09-24

**Authors:** Katharina Maria Lichtenegger, Lara Scholle-Winkler, Julia Kopanz, Angela Libiseller, Klaus Donsa, Felix Aberer, Thomas Rudolf Pieber, Julia Katharina Mader

**Affiliations:** 1 Division of Endocrinology and Diabetology, Medical University of Graz, Auenbruggerplatz 15, Graz, Styria, 8036, Austria, 43 31638572766; 2 Joanneum Research Forschungsgesellschaft mbH, Graz, Styria, Austria

**Keywords:** diabetes management, diabetes mellitus, digital decision support system, home care service, implementation science, digital health

## Abstract

**Background:**

Older adults with diabetes are vulnerable, facing multimorbidity and challenges in reaching glycemic targets. When insulin therapy is required, limited knowledge of geriatric therapeutic goals often leads to regimens that increase the risk of hypoglycemia. Simplified approaches such as once-daily basal insulin are recommended, yet titration protocols and unclear regimens remain barriers in home care nursing. Digital decision support systems (DDSSs) such as GlucoTab address this gap by offering evidence-based titration guidance integrated into nursing workflows. DDSSs also strengthen nurse autonomy, which leads to improved care processes and better outcomes in home care settings.

**Objective:**

This study assessed the effects of a nurse-guided intervention including a DDSS for diabetes management in older adults with type 2 diabetes receiving home care nursing.

**Methods:**

The prospective quasi-experimental feasibility and implementation study was conducted using GlucoTab for diabetes management as part of a multicomponent intervention at 2 home care nursing sites. Nine adults with type 2 diabetes receiving insulin therapy were enrolled (n=5 women; mean age 77, SD 10 years; mean BMI 27.7, SD 4.7 kg/m^2^; mean baseline hemoglobin A_1c_ 59.5, SD 12.8 mmol/mol). After receiving training, nurses implemented the DDSS, which integrates evidence-based diabetes management recommendations for home care and nursing workflows. Diabetes-related hospital admissions and glycemic outcomes were assessed before, during, and after implementation of the DDSS intervention.

**Results:**

Before implementation, 5 diabetes-related emergency admissions led to 4 hospitalizations, compared with none during implementation and 4 after implementation, mostly due to hyperglycemia. The median hospital stay was 9 (IQR 8-15) days (before and after implementation). Diabetes management shifted from predominantly premixed insulin therapy at baseline to basal insulin with or without bolus insulin during implementation. Diabetes-related home care visits decreased from 1.81 to 1.01 per day (before vs during implementation), and general practitioner contacts for insulin adjustments declined (19 before implementation vs 16 after implementation). Mean morning blood glucose decreased from 212 (SD 72) mg/dL to 162 (SD 42) mg/dL, and the proportion of measurements within the target range (70‐180 mg/dL) increased from 39.0% to 70.8% before vs during implementation. No incidence of hypoglycemia requiring third-party help was observed during implementation. Adherence of nurses to DDSS recommendations exceeded 94%.

**Conclusions:**

This study suggests that integrating a DDSS in a multicomponent intervention into home care nursing yielded longitudinal changes in diabetes outcomes. Emergency visits and hospitalizations were lower during implementation, no severe hypoglycemia was observed, glycemic control improved throughout the observation period, and therapy was simplified in routine care. Nursing visits decreased, suggesting potential improvements in care processes and resource use. High adherence and acceptance by nurses indicate feasibility and usability in home care practice. These findings align with evidence from hospitalized patients and telehealth studies, suggesting the potential for clinical, safety, and efficiency benefits.

## Introduction

### Background

Worldwide, approximately 422 million people live with diabetes, most residing in low- and middle-income countries. The prevalence of diabetes has been steadily increasing across all income levels over recent decades [1]. The most common form is type 2 diabetes, accounting for more than 90% of all cases worldwide, with prevalence rising further in geriatric populations due to increasing life expectancy. In this context, Europe represents one of the regions with the highest diabetes prevalence worldwide, with a substantial and growing proportion of older adults affected, placing increasing pressure on health care systems and home-based care services [2].

### Complexity of Diabetes Management

Diabetes is a chronic, metabolic disorder characterized by elevated blood glucose levels and can be accompanied by a range of complications, including microvascular and macrovascular damage, as well as a multitude of common comorbidities (eg, hypertension, obesity, cardiovascular disease, and chronic kidney disease) [3]. Older adults in particular face an increased risk of adverse health outcomes due to comorbidities and often fail to achieve age-appropriate glycemic goals [4]. Clinical evidence confirms that effective blood glucose management can reduce diabetes-related complications [5]. The American Diabetes Association recommends simplified treatment regimens for older adults, emphasizing hypoglycemia prevention as a critical factor of individualized glycemic targets. With the aging population, more older adults will live with diabetes, and age-related declines in self-management skills and memory may further complicate care. Basal insulin regimens, often administered via prefilled pens once daily, are especially suitable for older adults due to their ease of use and low hypoglycemia risk. Despite the availability of modern antihyperglycemic agents, some individuals with type 2 diabetes will ultimately require and benefit from insulin therapy due to decreasing endogenous insulin secretion. Evidence from hospitalized patients with type 2 diabetes has shown that a basal-bolus regimen is clearly superior to premixed insulin, resulting in fewer hypoglycemic events at comparable mean blood glucose levels [5,6].

### Empowering Nurses in Diabetes Care

Older adults with diabetes in home care nursing frequently do not receive optimal diabetes management due to lack of information, support, and professional cohesion [7,8]. Effective chronic diabetes management demands an interdisciplinary approach in which nurses represent a leading, empowered player. Enhancing nurses’ clinical autonomy, confidence, access to up-to-date information, and appropriate training is essential to enable this role [9]. Nurse-led care models have consistently shown to improve the outcomes of people with chronic diseases such as diabetes. Outcomes also include improved quality of care, better chronic disease management, and reduced health care use in primary care settings [10]. Evidence indicates that nurses often spend a great deal of time engaging with individuals, enabling more comprehensive assessment and support [11]. Systematic reviews further show that nurse-led interventions are associated with higher patient satisfaction; reductions in hospitalizations; and, in some cases, even lower mortality [12]. Additionally, care delivered by well-trained nurses can be as effective or even superior to that provided by primary care physicians [13].

### Digital Decision Support Systems

Innovative point-of-care digital decision support systems (DDSSs) are increasingly recognized as useful tools to facilitate evidence-based care specifically in people of older age and with impaired health status and more pronounced comorbidity profiles [14,15]. Evidence from randomized controlled trials has demonstrated that technology-supported and digitally enabled care interventions can improve clinical outcomes in chronic diseases, including diabetes [16]. Similarly, telehealth and structured chronic disease management approaches have been shown to improve care processes and reduce health care use [17]. Developments in glycemic care include computerized order sets, blood glucose data networks with visualization, case-finding tools, insulin calculators, electronic treatment protocols, titration algorithms, and alert systems [18,19]. For example, the DDSS GlucoTab supports basal-bolus insulin titration workflows by structuring care processes and offering standardized recommendations in the hospital setting. While such systems are often well established in hospitals [20-22], it remains unclear whether these tools can be effectively used in home care nursing. Although evidence from hospital and telehealth settings supports the effectiveness of such digital interventions, their transferability to home care nursing environments remains underexplored [17]. Each tool must be tested in its new care setting as it cannot be assumed that the systems designed for hospitals will function seamlessly in home care nursing.

To address this gap, DDSSs designed for nursing care are gaining more and more attention. A recent review highlights the importance of integrating nurses into every stage of innovation design and implementation to preserve care-centric perspectives [23]. Current desktop tools and mobile apps for the nursing care of older adults use nursing process frameworks to personalize interventions, showing a trend toward smart, home-based support systems [24]. These findings align with broader themes of nurse empowerment and digitally supported, person-centered care.

### Objectives

This study aimed to assess the effects of a multicomponent intervention including a DDSS on diabetes management by assessing diabetes-related glycemic management outcomes and hospital admissions in older adults with type 2 diabetes receiving home care nursing. This study was guided by the following research questions: (1) How does the implementation of a nurse-led DDSS affect glycemic outcomes across pre-, during, and postimplementation phases? (2) How does the DDSS influence diabetes-related hospital admissions in older home-cared adults? Outcomes were examined over 3 phases: 6 months before implementation, 3 months during implementation, and 6 months after implementation of the nurse-guided DDSS for insulin therapy.

## Methods

### Overview

This study was conducted and reported as a prospective quasi-experimental feasibility and implementation study in a real-world setting. Reporting followed the STROBE (Strengthening the Reporting of Observational Studies in Epidemiology) guidelines where applicable given the nonrandomized observational design. Relevant elements from the CONSORT-AI (Consolidated Standards of Reporting Trials–AI) extension were considered for the description of the DDSS [25]; however, the CONSORT-AI was not fully applicable due to the nonrandomized, nontrial design of this study. Furthermore, GlucoTab (decide Clinical Software GmbH) is a rule-based clinical decision support system and not a machine learning–based AI system.

**Figure 1.**
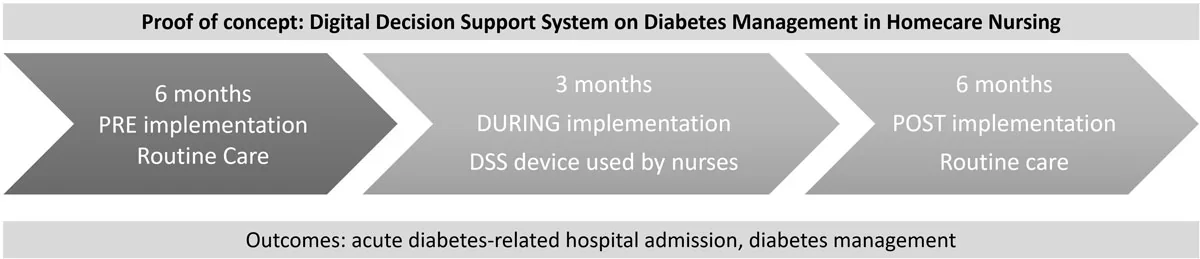
Study design to assess outcomes in the pre-, during, and postimplementation phases of a digital decision support system (DDSS) for diabetes management in home care nursing.

Following the overall study design, assessments were conducted at 3 predefined time points: before implementation (6 months), during implementation (implementation phase with active DDSS use; 3 months), and after implementation (postimplementation follow-up phase after DDSS discontinuation; 6 months; Figure 1). This study was conducted in Graz, Austria, at 2 home care nursing sites of the Austrian Red Cross, a private, nonprofit organization.

### Participants

A total of 9 individuals with type 2 diabetes were enrolled in the study. All 9 participants were assessed before implementation and during implementation. Only 8 participants were assessed after implementation as 1 participant died in the postimplementation phase.

Eligible participants were adults with type 2 diabetes treated with insulin therapy who received home care nursing from the Austrian Red Cross. Recruitment took place at 2 Austrian Red Cross home care sites. Local home care teams identified patients who met the predefined eligibility criteria and informed the study team about potentially eligible individuals. The most important exclusion criteria were medical conditions interfering with study participation or safety, other forms of diabetes (eg, type 1 diabetes), mental incapacity to provide consent, or terminal illness.

### Study Intervention

The intervention involved a multicomponent restructuring of care delivery, including structured nursing training, a DDSS, physician-guided initiation of insulin therapy, and standardized insulin titration protocols. The DDSS used (GlucoTab) included decision support for health care professionals regarding automated suggestions for insulin dose, adjustments for basal insulin therapy, and changes in the insulin regimen. The DDSS also included suggestions for blood glucose measurement frequency, workflow support through visualization of open tasks, and support with a documentation interface. In a previous study, the basal insulin algorithm was specifically adapted for geriatric individuals [26]. An evidence-based version developed in line with international guidelines [3] was digitally implemented integrated into the GlucoTab software, with the system and workflows tailored to the routines of home care nursing. Importantly, the system empowers nurses to act directly in the individual’s home based on current blood glucose values independently of physician availability, including evenings and weekends, when medical support is often limited.

Before implementation, home care nurses and supervising study physicians participated in a structured group training covering the intervention.

During the implementation phase of the intervention, the study physicians prescribed the initial insulin regimen, and subsequent dosing was managed by home care nurses using GlucoTab. The DDSS allows physicians to either independently determine the initial basal insulin dose for participants (already receiving insulin therapy) or request an algorithm-generated dosing recommendation for participants newly initiated on insulin therapy. In both cases, the final decision on initial insulin therapy remains physician led. GlucoTab automatically flags unsafe situations (eg, low blood glucose values) and ensures physician review when necessary. The DDSS automatically records all generated recommendations and corresponding nursing actions, enabling objective and reproducible calculation of adherence rates [20-22]. The system uses a mobile client-server architecture connected via a secure Long Term Evolution network to a centralized, secure server environment hosted on the premises of Joanneum Research Forschungsgesellschaft mbH (Austria).

During the implementation phase, nurses accessed GlucoTab via standard tablet devices using individual secure log-ins during home care visits. The system does not require dedicated medical hardware and was used directly at the point of care to document blood glucose values, insulin administration, and relevant clinical information. On the basis of the entered data, GlucoTab generated individualized, algorithm-based decision support recommendations for insulin management. Nurses reviewed these recommendations and integrated them into their clinical decision-making process while retaining responsibility for the final insulin dosing and administration decision.

During the pre- and postimplementation phases, participants received routine care, which encompassed paper-based diabetes management, including diabetes therapy, and the frequency of blood glucose measurements was prescribed by the treating general practitioner. Home care nurses performed blood glucose monitoring and administered diabetes therapy as prescribed. Importantly, the postimplementation phase represents routine care after DDSS discontinuation rather than a genuine return to baseline conditions as training effects and workflow changes established during implementation may persist into the postimplementation phase.

### Assessments and Data Analysis

In all phases, clinical and demographic data and diabetes management data (blood glucose values, measurement frequency, insulin dosing and injection, general practitioner contacts, and diabetes-related hospital admissions) were collected from (electronic) health records and paper-based nursing documentation of the home care nursing provider.

Hypoglycemia was defined as capillary blood glucose values below 54 mg/dL and/or hypoglycemic events requiring third-party assistance. Hypoglycemic events were identified from nursing documentation and blood glucose records. Continuous glucose monitoring was not available in this study.

During the implementation phase, additional data regarding diabetes management were recorded directly in the DDSS for nurses. Clinical and demographic data were digitally entered into electronic case report forms (OpenClinica; OpenClinica, LLC) and spreadsheets by a qualified researcher. Quality control was ensured by applying a 4-eye principle to verify completeness and accuracy.

Nurse acceptance was operationalized as adherence to DDSS-generated recommendations and was objectively measured using automatically logged system data capturing nurse responses and actions within the DDSS.

Data analysis was performed using the statistical program SPSS Statistics (version 26; IBM Corp) and Microsoft Excel. Data were first summarized using descriptive statistics. For numerical data, depending on the distribution, means and SDs; medians; minimum, maximum, and range; and quartiles were calculated. Categorical data are presented as absolute and relative frequencies. Due to the quasi-experimental feasibility and implementation design of the study, no inferential statistical analyses and no sample size calculation were conducted.

### Demographics

Nine participants receiving home care nursing were included (n=5 women; mean age 77, SD 10 years; mean hemoglobin A_1c_ [HbA_1c_] 59.5, SD 12.8 mmol/mol; mean BMI 27.7, SD 4.7 kg/m^2^ [before implementation]). Before implementation, 4 participants had diabetes-related comorbidities: retinopathy (n=2), neuropathy (n=3), and nephropathy (n=3).

The 9 participants completed all 3 study phases as per the protocol. One participant was hospitalized (unrelated to the study) and withdrew but was included in the analysis as intervention duration was completed. One participant died during the postimplementation phase for reasons unrelated to the study.

### Ethical Considerations

This study was approved by the Ethics Committee of the Medical University of Graz (approval numbers EK 30‐287 ex 17/18 and EK 31‐522 ex 18/19) and relevant legal authorities. It was conducted in compliance with good clinical practice and the principles of the Declaration of Helsinki.

All eligible patients identified were invited to participate; all provided informed consent and were enrolled in the study. To ensure confidentiality, participants were assigned unique study identification numbers, preventing traceability of personal data. No participant compensation was provided.

## Results

### Diabetes-Related Hospital Admissions

During the preimplementation phase, 5 diabetes-related emergency admissions were documented in 4 participants, resulting in 4 hospitalizations. In the postimplementation phase (after DDSS discontinuation), 4 diabetes-related emergency admissions occurred in 2 participants, leading to 1 referral to the diabetes outpatient clinic and 2 hospitalizations. No diabetes-related emergency admissions or hospitalizations were observed during the implementation phase (Table 1).

**Table 1.** Diabetes management and outcomes before, during, and after implementation of the GlucoTab system.

	Routine care (6 months before implementation; n=9)	Decision support (3 months during implementation; n=9)	Routine care (6 months after implementation; n=8^a^)
Insulin therapy, n (%)			
Premixed insulin	5 (55.6)	0 (0)	0 (0)
Premixed insulin+bolus	2 (22.2)	0 (0)	0 (0)
Basal insulin	1 (11.1)	9 (100)	5 (62.5)
Basal insulin+bolus^b^	1 (11.1)	0 (0)	3 (37.5)
Hospital admissions, n	5	0	4
Inpatient stays among admissions	4	0	2
Morning capillary blood glucose (mg/dL), mean (SD)	212 (72)	Month 1: 171 (68); month 2: 150 (46); month 3: 145 (35)	162 (42)
Blood glucose values by range (mg/dL), % (n/N)^c^			
<54	0.1	0.0 (0/992)	0.0
<70	0.2	0.3 (3/992)	0.4
70-180	39.0	77.0 (764/992)	70.8
>180	60.8	22.1 (219/ 992)	28.8
HbA_1c_^d^ (mmol/mol), mean (SD)	59.5 (12.8)	56.5 (12.4)	—^e^

a One participant died during the postimplementation phase.

b Bolus insulin for main meal or for correctional insulin.

c Absolute frequencies (n/N) cannot be provided for before and after implementation because the original dataset is currently unavailable.

d HbA_1c_: hemoglobin A_1c_.

e HbA_1c_ was not available in the postimplementation phase.

Hyperglycemia was the reason for referral in 8 of the 9 diabetes-related emergency admissions; the remaining diabetes-related emergency admission was due to hyperglycemia. The length of inpatient stays ranged from 8 to 15 days, with a median of 9 days.

### Nurse-Led Diabetes Processes in Home Care

Table 1 summarizes diabetes management and outcomes across the 3 study phases. In the preimplementation phase, 77.8% (7/9) of the participants received premixed insulin, including 22.2% (2/9) with additional bolus insulin. A total of 22.2% (2/9) of the participants received basal insulin, of whom 1 also received bolus insulin. After study completion, all participants (8/8, 100%) were treated with basal insulin, with 37.5% (3/8) additionally receiving bolus insulin for correcting hyperglycemia. In 62.5% (5/8) of the participants, the suggested basal insulin titrations were implemented by general practitioners; in 37.5% (3/8) of the participants, the recommended corrections were not adopted during the postimplementation phase.

During the implementation phase, therapy adjustments based on DDSS recommendations were made on average 9 times in the 3 months (up to 18 times). The number of diabetes-related home care nursing visits decreased from 1.81 to 1.01 per day when comparing the pre- and during implementation phases, largely due to reduced necessity of blood glucose checks.

In total, 35 diabetes-related contacts with general practitioners were documented, of which 19 (54.3%) occurred in the 6-month preimplementation phase and 16 (45.7%) occurred in the postimplementation phase, indicating a slight reduction after the implementation phase. In the preimplementation phase, 36.8% (7/19) of the general practitioner contacts were due to a planned checkup, 57.9% (11/19) were for therapy adjustment, and 5.3% (1/19) were for HbA_1c_ determination. In the postimplementation phase, 31.3% (5/16) of the general practitioner contacts were due to planned controls, and 68.8% (11/16) were due to therapy adjustment. During the implementation phase, nurses contacted the outpatient diabetes clinic via telephone as suggested by the DDSS for receiving medical advice, but no quantitative data were systematically recorded in this phase.

### Glycemic Outcomes

Mean morning blood glucose values decreased from 212 (SD 72) mg/dL before implementation to 162 (SD 42) mg/dL after implementation. No hypoglycemic events requiring third-party assistance were observed during any study phase. Biochemical hypoglycemia (<54 mg/dL) was rare, with 0.1% of glucose measurements below this threshold in the preimplementation phase and no values below 54 mg/dL during implementation or after implementation. The proportion of blood glucose measurements within the prespecified target range (70‐180 mg/dL) was 39.0% before implementation, 77% (764/992) during implementation, and 70.8% after implementation, whereas measurements above 180 mg/dL were 60.8%, 22.1% (219/992), and 28.8%, respectively (n/N values were only available for the during-implementation period).

**Figure 2.**
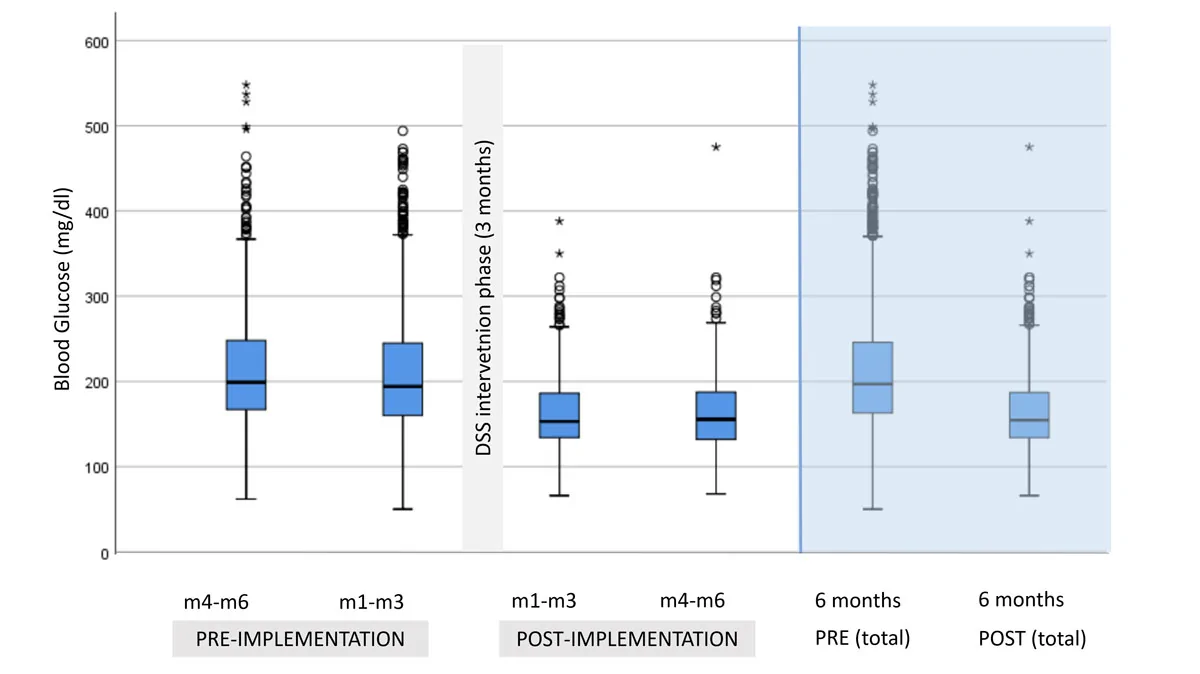
Box plots for blood glucose values in the pre- and postimplementation phases (boxes represent the IQR with the median, whiskers indicate 1.5 times the IQR, dots indicate outliers [1.5‐3 times the IQR], and stars indicate extreme outliers [>3 times the IQR]). The figure shows longitudinal glucose measurements over time, with repeated measurements from the same participants reflecting real-world glucose monitoring. DSS: decision support system.

The distribution of blood glucose values over time showed a clear trend toward improved glycemic control (Figure 2, right panel). Higher mean values and wider variability were observed in the preimplementation phase. In contrast, a marked reduction in median blood glucose levels and variability was found in the implementation phase. This improvement was sustained during the following 3 months, with the 6-month postimplementation phase showing improved glycemic control compared to the 6-month preimplementation phase.

### User Acceptance

In 88.9% (8/9) of cases, physicians chose to set the initial basal insulin dose independently without using the DDSS-generated recommendation (all participants already received insulin therapy before GlucoTab implementation). Adherence to the DDSS recommendations by nurses was high: 94.7% (552/583) for blood glucose measurement frequency, 95.8% (295/308) for basal insulin dose adjustment, 99.7% (764/766) for basal insulin injections, 97.9% (142/145) for bolus insulin injections, and 100% (231/231) for titration time points.

## Discussion

### Principal Findings

This prospective quasi-experimental feasibility and implementation study suggests that integration of a DDSS into home care nursing corresponded with improved diabetes management outcomes over time. During the implementation of the DDSS, emergency department visits and hospitalizations were reduced, highlighting improved patient safety and favorable clinical outcomes. Glycemic control improved over time, while therapy appeared simplified and nursing visits decreased, suggesting potential improvements in efficiency and resource use.

Diabetes management in home care or long-term care facilities is often challenged by the complexity of insulin regimens used in older adults, the high variability of daily blood glucose levels, and the frequent need for medical decisions. Many older adults with diabetes receive once- or twice-daily basal or premixed insulin, which still requires regular dose adjustments [8,27]. However, in clinical practice, contacts with general practitioners occur infrequently, dose adaptations are often delayed, and nurses are frequently forced to act autonomously without formal medical orders. As a consequence, clinical uncertainties can lead to avoidable emergency department transfers or hospital admissions [8]. DDSSs directly address these challenges by providing structured, algorithm-guided titration that supports timely and safe decisions at the point of care.

Our study findings extend the evidence base for algorithm-guided diabetes management using GlucoTab as initially demonstrated in hospitalized patients [20-22], where the decision support system guided insulin dosing, achieving effective glycemic control with reduced hypoglycemia and appropriate adherence to dose suggestions. A recent comprehensive review emphasizes that technological solutions, including connected insulin pens, continuous glucose monitoring, automated insulin delivery systems, and other digital tools, play an increasingly important role in the care of older adults with diabetes. Through reduction of blood glucose variability and hypoglycemia risk, as well as by improving quality of life and reducing hospital admissions, such technologies support safe and effective long-term diabetes management in older, multimorbid populations [15]. This broad evidence for diabetes technology in older adults reinforces the relevance and potential impact of GlucoTab in home care nursing.

Beyond home care, randomized controlled trials have shown that nurse-assisted insulin titration can safely improve glycemic control. For example, decision-supported telephone coaching by diabetes specialty nurses enabled individuals to self-titrate basal insulin safely, resulting in greater HbA_1c_ reductions without increasing hypoglycemia compared to usual care [28]. This previous study underscores that combining decision support with nurse engagement meaningfully improves glycemic outcomes in noninstitutionalized individuals, supporting our findings from this quasi-experimental feasibility and implementation study in home care nursing.

The high acceptance levels that we observed among nurses, with adherence rates exceeding 94%, align with nursing-focused digital health literature. These findings are in line with evidence that nurse-led care models improve chronic disease outcomes and strengthen care quality in primary care settings [10]. Recent studies published in *JMIR Nursing* and *JMIR Diabetes* underscore the importance of implementing user-friendly digital tools in fostering adherence and enabling nurse-led care innovations [29,30]. Usability is a key concept in the evaluation of digital health technologies and can be defined as the extent to which a system enables users to achieve specified goals with effectiveness, efficiency, and satisfaction. Importantly, usability is influenced by the users, tasks, and context of use, which is particularly relevant in home care settings where clinical decision-making occurs in complex real-world environments [31]. Established usability evaluation methods include inspection-based approaches and user-centered evaluation techniques that are used to identify usability problems and barriers to system use during development and implementation [32]. However, formal usability testing was not conducted in the present study. Recent digital health research further emphasizes that successful implementation of health technologies requires the combined evaluation of usability, user experience, and implementation outcomes as these factors jointly influence adoption, safety, and long-term sustainability [33]. This supports the notion that the DDSS not only augments clinical outcomes but also empowers nursing autonomy and confidence, allowing nurses to integrate the DDSS seamlessly into daily routines and care activities and spend more time on direct patient care rather than administrative tasks or frequent coordination with general practitioners.

The observed reduction in home nursing visits and the simplification of therapy processes through GlucoTab suggest considerable potential for cost savings and efficiency gains in home care nursing services. At the same time, the DDSS strengthens nurses’ autonomy and supports safe, evidence-based decision-making without continuous physician oversight. This is in line with international guidelines advocating for structured nurse-led insulin titration under standardized protocols (eg, the Irish national insulin titration guideline for nurses) [34]. These effects align with broader health system goals of optimizing resource use without compromising quality, a particularly relevant consideration in international settings with varying home care constraints. Similar findings have been reported in other studies. A systematic review demonstrated that telehealth interventions for communication between individuals with type 2 diabetes and health care professionals significantly improved glycemic control, with 80% of included studies also showing potential for cost reduction [35]. Another study reported a reduction in overall health care costs of approximately US $24,000 over 7 months through telehealth-based diabetes management, highlighting substantial savings for the health care system [36].

Recent advances in AI and digital health research further contextualize these findings. Emerging AI-enabled decision support prototypes for basal insulin titration have shown promising usability and safety in hospital and early primary care evaluations, indicating that nurse-in-the-loop models could further enhance precision and individualization of insulin therapy in home care settings. Integrating such tools may help reduce practice variation, strengthen guideline adherence, and potentially open pathways for decision support in broader chronic disease management beyond insulin therapy [24,37-39].

These findings suggest the potential of digital interventions such as GlucoTab not only to improve clinical outcomes but also to enhance efficiency and reduce costs in home care nursing services by minimizing the need for frequent coordination with general practitioners, enabling nurses to work more autonomously with decision support, and reducing dependence on continuous medical orders.

### Limitations and Future Directions

The relatively small sample size and observational design may limit the generalizability of our findings. The observational pretest-during-posttest design without a control group limits causal inference. All outcomes should be interpreted as descriptive and hypothesis generating rather than causal evidence of effectiveness. A key limitation is that the postimplementation phase does not reflect a return to baseline as participants and care processes may have been influenced by DDSS implementation, structured training, and persistent workflow adaptations. Pretest-posttest comparisons should thus not be interpreted as changes between fully independent states.

The variation in the lengths of the study phases (6-3-6 months) and the limited external validity due to participation of only two study sites in a single region also constrain the transferability to other settings. Long-term sustainability of the observed improvements, therefore, requires confirmation through larger, ideally randomized studies conducted across diverse health care systems.

Future research should focus on (1) evaluating cost-effectiveness through detailed economic analyses; (2) testing scalability and adaptation of GlucoTab in varied international home care models; and (3) exploring the effects of DDSSs on broader populations, including non–insulin-dependent individuals with type 2 diabetes or other chronic diseases and further patient-centered clinical end points (eg, mortality and disability). Furthermore, glycemic control might be more intensively supervised by using (blinded) continuous glucose monitoring systems in future studies to obtain a broader picture of the efficacy of such a DDSS.

### Relevance to Clinical Practice for the Wider Global Clinical Community

This study provides in-depth insights into the quality of diabetes management for people receiving home care nursing, a population that is often underrepresented in research. It generates evidence to guide policymakers and nursing care managers in designing strategies that ensure high-quality diabetes care in home care settings by strengthening the role and competencies of nursing staff. Furthermore, it presents an exemplary digital decision support solution that empowers nurses, enhances the quality of care, and promotes interdisciplinary collaboration with general practitioners, thereby offering a transferable model for other health care systems.

### Conclusions

In the long term, DDSSs have the potential not only to reduce costs in the home care setting but also to enhance the evidence-based quality and safety of care for people with type 2 diabetes. This quasi-experimental feasibility and implementation study provides the first evidence that a nurse-led DDSS such as GlucoTab can safely and effectively improve glycemic control in older adults receiving home care nursing, reduce hospitalizations, simplify therapy, and enhance nurse autonomy. Given its feasibility, this approach could empower nurse-led diabetes management, optimize resource use, and improve outcomes for people with diabetes in diverse health care settings. Collectively, these findings highlight the potential of digitally supported, nurse-driven interventions as scalable, patient-centered solutions for managing complex diabetes care in community and long-term care environments.

## Abbreviations

CONSORT-AI: Consolidated Standards of Reporting Trials–AI
DDSS: digital decision support system
HbA_1c_: hemoglobin A_1c_
STROBE: Strengthening the Reporting of Observational Studies in Epidemiology
